# In Utero Electroporation for Manipulation of Specific Neuronal Populations

**DOI:** 10.3390/membranes12050513

**Published:** 2022-05-11

**Authors:** Kotaro Yamashiro, Yuji Ikegaya, Nobuyoshi Matsumoto

**Affiliations:** 1 Graduate School of Pharmaceutical Sciences, The University of Tokyo, Tokyo 113-0033, Japan; kyamashiro7@gmail.com (K.Y.); yuji@ikegaya.jp (Y.I.); 2 Institute for AI and Beyond, The University of Tokyo, Tokyo 113-0033, Japan; 3 Center for Information and Neural Networks, National Institute of Information and Communications Technology, Osaka 565-0871, Japan

**Keywords:** in utero electroporation, channelrhodopsin, optogenetics

## Abstract

The complexity of brain functions is supported by the heterogeneity of brain tissue and millisecond-scale information processing. Understanding how complex neural circuits control animal behavior requires the precise manipulation of specific neuronal subtypes at high spatiotemporal resolution. In utero electroporation, when combined with optogenetics, is a powerful method for precisely controlling the activity of specific neurons. Optogenetics allows for the control of cellular membrane potentials through light-sensitive ion channels artificially expressed in the plasma membrane of neurons. Here, we first review the basic mechanisms and characteristics of in utero electroporation. Then, we discuss recent applications of in utero electroporation combined with optogenetics to investigate the functions and characteristics of specific regions, layers, and cell types. These techniques will pave the way for further advances in understanding the complex neuronal and circuit mechanisms that underlie behavioral outputs.

## 1. Introduction

Optogenetics has become a powerful tool in neuroscience for understanding the complex functions of neural networks [1,2]. In the past decade, the contribution of specific neuronal populations to complex neuronal dynamics and their effect on behavior have been revealed using optogenetic approaches [3,4,5]. By expressing light-gated ion channels and pumps in distinct neuronal populations, researchers can precisely control the activity of specific neurons [2,5,6,7]. Optogenetic approaches have enabled the exploration of a variety of neuronal populations, revealing a series of neuronal circuits associated with sensory processing, memory, and learning [8,9,10,11,12,13].

Neurons, like all other cells, have a plasma membrane. The plasma membrane serves as a barrier between the neuron and its surroundings, controlling the movement of substances into and out of the cell. This movement is controlled by transmembrane proteins (embedded in the plasma membrane), which act as ion channels or pumps that control the transport of specific molecules across the membrane. Electrically charged ions are one of the substances transported by ion channels and pumps. Ion channels and pumps transport specific ions and generate voltage gradients across the membrane, which are referred to as membrane potentials. In particular, a rapid influx of sodium ions generates action potentials, which contribute to neuron-to-neuron communication in the following manner. in neurons, action potentials are conducted along axons toward synaptic boutons at presynaptic axon terminals, where the electric signals are converted into chemical signals (e.g., Glu (glutamic acid) and GABA (gamma-aminobutyric acid) in most cases). The neurotransmitters (i.e., Glu or GABA) bind to ion channel-coupled receptors on postsynaptic terminals at chemical synapses, which transform these chemical signals back into electric signals known as postsynaptic excitatory or inhibitory potentials. Collectively, the generation of ac-tion potentials and the subsequent activa-tion of postsynaptic neurons are achieved by a number of ion channels.

In the field of optogenetics, researchers often genetically modify neurons to manipulate their activity by inducing their expression of light-gated ion channels or pumps known as opsins [3,14,15,16]. Opsins, which are often used in optogenetics, are channels or pumps that transport specific ions upon absorbing specific light frequencies [3,14,17,18,19,20,21,22,23]. When opsin-expressing neurons are stimulated with light, light-gated channels are activated, which either promote or inhibit the generation of action potentials [17,18,20,21,23].

Among the various methods for promoting the expression of opsins in a specific neuronal population, we focus on in utero electroporation, which allows researchers to study the diversity of the neuronal populations composing neural circuits [24,25,26]. This review aims to improve the understanding of in utero electroporation and consider how to exploit its potential in combination with optogenetics.

First, we introduce the unique features of the technique by comparing it with similar techniques. Then, we precisely illustrate the basic function and characteristics of in utero electroporation. Finally, we discuss future perspectives by showing examples of previous studies that have combined in utero electroporation with optogenetics to reveal important features of neural circuits.

## 2. Pros and Cons of Several Methods for Expressing Opsin in Neurons In Vivo

**Figure 1:**
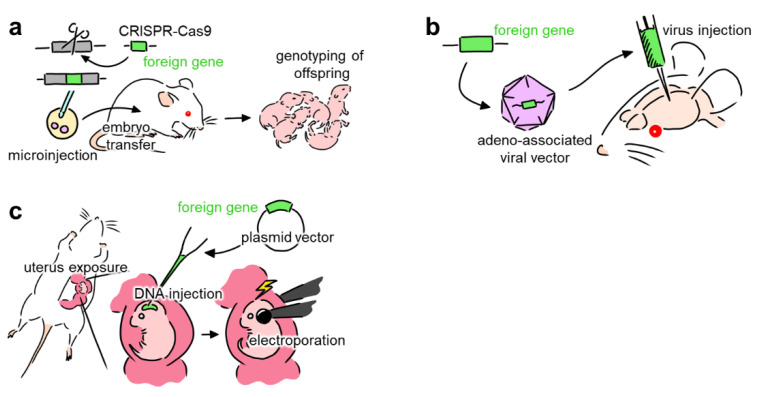
Overview of methods that introduce foreign genes into neurons. (**a**) Schematic of creating genetically modified animals that express opsins. The DNA sequence of the target animal is modified and injected into embryos. The embryos are then transferred to the host animal, where they mature into offspring. (**b**) Schematic of transduction enabling the expression of opsins using viral vectors. DNA segments with foreign genes are encapsulated into a viral vector. After injecting the viral vectors into a specific target region of the brain, the virus transfects neurons in that region, inducing opsin expression. (**c**) Schematic of in utero electroporation. Using laparotomy, the uterus of the pregnant animal is exposed. Then, DNA plasmid vectors with the target genes are injected into the ventricle of the fetus. Electroporation is then used to transfer the DNA plasmids into the cell.

Neural networks comprise a myriad of cells, each receiving and sending projections to various brain regions. Thus, cellular- and projection-specific optogenetic control of neural activity is required to understand the complex network structure of neurons. Several methods, including (i) genetically modified animals (Figure 1a), (ii) viral vectors (Figure 1b), and (iii) in utero electroporation (Figure 1c), have been used to promote the expression of opsins in the neurons of living animals [27].

### 2.1. Genetically Modified Animals

Genetically modified animals can be created by altering their genetic materials (i.e., DNA in most cases) with genetic engineering techniques. Conventional genetic engineering methods allow researchers to insert ‘foreign’ genes at a random location in the genome of a host animal to create transgenic animals. However, conventional genetic engineering methods do not necessarily yield the desired outcomes because foreign genes are inserted at unpredictable loci in the host animal’s genome. In this light, this approach has recently been described as ‘ham-fisted’ or clumsy [28].

In addition to the conventional methods described above, gene editing technologies based on the zinc finger nuclease (ZFN) [29,30,31,32,33,34], transcription activator-like effector nuclease (TALEN) [30,35,36,37,38,39,40,41], and clustered regular interspaced short palindromic repeats-Cas9 (CRISPR–Cas9) [30,35,42,43,44,45] systems have recently been developed, allowing researchers to precisely modify the DNA sequence of host animals. These systems allowed for gene targeting by inactivating specific genes (known as a knockout) or inserting a foreign DNA sequence into a gene of interest (known as knock-in). Among the various knockout and knock-in animals, mice that express Cre recombinase under a specific promoter are widely used. Crossing a Cre mouse line with another strain carrying Cre-dependent opsins limits the expression of opsins to Cre-expressing cells.

Another method uses a viral vector with opsin genes (see the next section). There are numerous Cre lines for specific neurons in mice, but the number of opsin-expressing mouse models is limited. Moreover, the drawback of this system is that genetically modified animals for species other than mice are meager and scarce. Theoretically, one could design and create novel transgenic or knock-in animal lines for various species, but this would be a time-consuming and costly process.

### 2.2. Viral Vectors

Using viral vectors for gene expression is an alternative to producing transgenic animals. Gene expression with viral vectors (e.g., adeno-associated virus (AAV) and lentivirus) is achieved by replacing the viral genome with a custom-designed construct containing a target opsin [46]. This technique enables precise spatial control as well as cell-type specificity through the use of specific promoters and enhancers. For example, universal promoters drive gene expression in a wide variety of neurons, while cell-type-specific promoters limit the expression of genes to specific types of neurons. Further cell-type specificity can be achieved by using trans-synaptic viral vectors to target specific projections. Retrograde- and anterograde-transporting viral vectors (e.g., rAAV2-retro [47], rabies virus (RV) [48], herpes simplex virus 1 (HSV-1) [49] and vesicular stomatitis virus (VSV) [50]) can be used to deliver target genes in a projection-specific manner. This strategy, combined with conditional expression machinery, enables the circuit-specific expression of opsins in various animal models. The disadvantage of this approach is the limited packaging capacity of viral vectors. AAV and lentivirus vectors can accommodate genomes with up to 5 kb and 10 kb of packaged transgene constructs, respectively. Due to this bottleneck, viral vectors can only be used to deliver relatively small promoters.

### 2.3. In Utero Electroporation

In utero electroporation is used to induce the expression of target genes in neural precursors to control the activities of neurons. This method is less invasive and more convenient than postnatal injections of viral vectors [51,52,53,54,55,56]. In most cases, the plasmid vector (to be expressed) is injected into the ventricles of the embryonic brain. Then, electrical pulses are used to transfer the DNA into cells in the adjacent ventricular zone or subventricular zone [55,56]. The target gene can be expressed in a specific neuronal population by controlling the embryonic day when the method is applied. The area of expression can also be controlled by placing bipolar electrodes at specific angles and positions [53,57,58,59,60]. Electroporation can be used in various animals, including mice, rats, ferrets, and cats [53,54,57,61,62]. Furthermore, electroporated cells contain multiple copies of the transgenes, resulting in a reliable and robust expression of the target genes. However, the drawback of in utero electroporation is the uncertainty in how long the transgenes are expressed. This is because electroporated plasmid vectors are likely to be episomal, and the expression of foreign genes depends on the total amount of the plasmid vectors. If nonviral vectors are introduced into proliferating cells and inherited in daughter cells, the number of vectors in each daughter cell is less than that in the mother cell because the total number of vectors (remaining episomally in the mother cell) is constant [63]. Due to this issue, without combining it with other techniques, such as transposon-mediated gene expression systems [64,65,66,67,68], in utero electroporation is not suitable for studying constantly proliferating cells, such as apical progenitors, basal progenitors, and outer subventricular zone (OSVZ) radial glia-like cells, all of which contribute to the generation of glutamatergic neurons as neural progenitor cells.

## 3. Principle of Electroporation

Electroporation consists of two major steps: (1) electropermeabilization of the cellular membrane and (2) electrotransfer of a DNA plasmid. Both steps in the electroporation process require a train of electric pulses to drive the DNA into target cells. The efficient transfer of plasmid DNA into the cells depends on (1) the permeability of the cell membrane and (2) the probability (i.e., success rate) of DNA transfer across the cell membrane toward the nuclear membrane. These can be controlled by optimizing the frequency and intensity of the electrical pulses. In the following section, we discuss the basic mechanisms of electroporation.

### 3.1. Electropermeabilization of the Cell Membrane

In the first step of electroporation, a series of electric pulses are used to disrupt the cell membrane, resulting in the formation of temporary pores in the membrane [69,70]. The formation of these pores is dependent on the increased transmembrane voltage caused by the applied electric field. The transmembrane potentials are temporarily elevated by multiple electric pulses, generating ion flow. This ion flow charges the membrane and causes a rapid rearrangement of the molecular structures in a confined space, forming pores in the membrane. Owing to this transient pore formation, hydrophilic molecules, including DNA, can be transported into and out of the cell. Upon removal of the external electric field, these pores rapidly close. Depending on the target tissues and the model animal, the voltage can range from 10 V to 1000 V [71]. However, a voltage of 20–50 V is typically used for in utero electroporation of the rat or mouse brain [58,72].

### 3.2. Electrotransfer of Plasmid DNA

In the second step, the negatively charged DNA migrates toward the positive electrode [73,74]. The process of transferring DNA into cells is equivalent to the process of using DNA electrophoresis in an external electric field. As previous studies have suggested, transfection efficiency is significantly higher in cathode-facing cell monolayers than in cells exposed to electric field pulses in the opposite direction (i.e., the anode-facing direction) [75,76]. This is because negatively charged plasmid DNA migrates through the electrically permeable cell membrane via electrophoresis. Therefore, as described in a later section, it is possible to physically control the transfection site by changing the position and polarity of the electric field.

## 4. Region-Specific Expression Using In Utero Electroporation

The brain comprises multiple functionally distinct regions, and proper neural circuits between these regions are required for higher brain functions. Various genetic modification techniques, such as the use of transgenic Cre lines and viral vectors, have been developed to induce the conditional expression of transgenes in specific neural populations.

**Figure 2:**
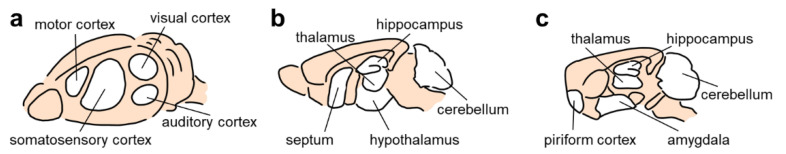
Regions that can be targeted using in utero electroporation. (**a**) Diagonal view of the rodent brain from the surface. (**b**) Sagittal section of the medial part of the rodent brain. (**c**) Sagittal section of the lateral part of the rodent brain.

In utero electroporation can be used to induce gene expression in certain brain regions by targeting progenitor cells that migrate and develop into neurons in specific brain regions (Figure 2). This is achieved by orienting the position and angle of the electrode while administering electrical pulses. Specific brain region targets include the motor cortex, prefrontal cortex, visual cortex, hippocampus, cerebellum, lateral septal nucleus, striatum, thalamus, and hypothalamus [53,57,62,77,78,79]. In particular, directed electroporation of the mouse or rat neocortex (i.e., the motor cortex, somatosensory cortex, visual cortex, and prefrontal cortex) is achieved by placing the positive electrode at dorsolateral positions, thereby promoting the transfer of the plasmid to cells in the dorsolateral region of the lateral ventricle [53,57]. Similarly, plasmids can be transferred to neural precursor cells in the hippocampus by placing the positive electrode on the medial region of the lateral ventricle [53,57,78]. Placing the positive electrode ventrolaterally promotes the transfection of the plasmid DNA into pyramidal neurons in the piriform cortex and amygdala [80].

**Figure 3:**
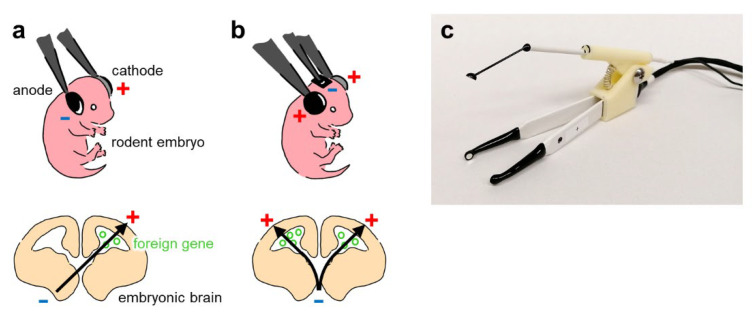
Double- and triple-electrode probe techniques for in utero electroporation. (**a**) Top: Electroporation using a double-electrode probe, with the anode and cathode indicated in blue and red, respectively. Bottom: The black arrow indicates the flow direction of plasmid DNA as DNA is negatively charged. (**b**) Top: Electroporation using a triple-electrode probe. Bottom: The triple-electrode probe technique enables the transfer of plasmid DNA into both the right and left ventricular zones. (**c**) Photograph of our custom-made tripolar-electrode probe and handle.

The bipolar in utero electroporation technique was first developed in 2001 [55,81] (Figure 3a). Initially, it was developed as a quick and simple method for genetically manipulating pyramidal neurons in the mouse somatosensory cortex in vivo. Over the next decade, more studies used the standard in utero electroporation technique in the somatosensory cortex. This technique was also used to transfect other brain regions, including the hippocampus and prefrontal cortex. However, since the in utero electroporation technique in those days mainly made use of double-electrode probes with two poles (i.e., bipolar), it was extremely difficult to perform transfection in areas other than the somatosensory cortex [58,72]. However, in 2012, dal Maschio et al. developed triple-electrode (i.e., tripolar) probes for in utero electroporation, which enabled the efficient transfection of regions other than the somatosensory cortex, including the hippocampus, prefrontal cortex, motor cortex, visual cortex, and cerebellum [58] (Figure 3b). In addition, triple-electrode probes enable researchers to create a symmetrical electric field, allowing gene transfer on both sides of the brain. Notably, prior to the development of triple-electrode probes, gene transfers were only possible on the contralateral side of the brain; if two-electrode probes were used, undesired transfection of the other ventricle could occur in the case of the plasmid loading of both ventricles. Moreover, portable electroporators for in utero electroporation have recently been developed [59].

Confinement of the transgene-expressing region is also a benefit of using in utero electroporation. As is often the case with viral vectors, simple diffusion after injection causes surrounding regions to also be transfected. However, since transfection of plasmids in in utero electroporation is also affected by the direction and intensity of the electric field, controlling these parameters will enable researchers to confine the region of transgene-expressing tissues. Although region-specific expression of transgenes is possible with double-electrode probes, the diffused electric field may cause the transfection of surrounding tissues, and an asymmetrical electric field results in an undesired transfection of the other ventricle when loading both ventricles with plasmid vectors. Undesired expression in other regions can be overcome by using triple-electrode probes to precisely regulate the direction and strength of the electric field [58]. Using a triple-electrode probe (Figure 3c), researchers can confine the transgene-expressing region to a very narrow area even if there are no specific genetic markers for the region.

## 5. Layer- and Cell Type-Specific Manipulation of Neurons

**Figure 4:**
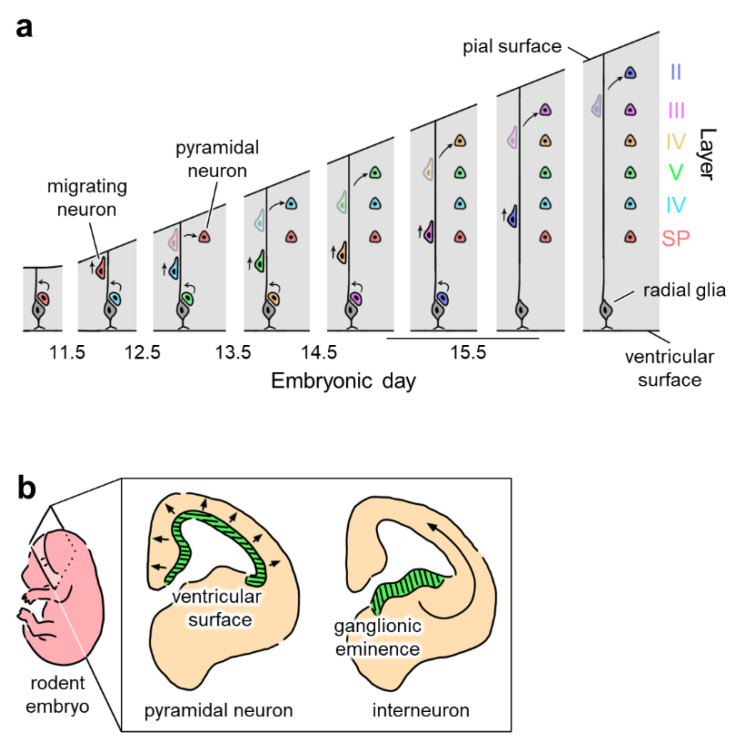
Region- and cell type-specific in utero electroporation by targeting precursor cells. (**a**) Inside-out scheme of neuronal migration (indicated by arrows) in the developing neocortical layers. The pyramidal neurons in deep layers of the cortex form earlier than pyramidal neurons in superficial layers. Pyramidal neurons in cortical layers II, III, IV, V, and VI and the subplate (SP) are indicated in blue, pink, yellow, green, light blue, and red, respectively. Radial glial cells in the ventricular zone are indicated in gray. Newborn neurons migrate into the cortex using radial glia spanning from the ventricular to the pial surface. Expression of foreign genes in a specific cortical layer can be achieved by in utero electroporation targeting newly born neurons that mature into pyramidal neurons in the target layer. (**b**) Origins of pyramidal neurons and interneurons. Pyramidal neurons generated in the ventricular surface (middle, green) migrate radially (in the direction of arrows), while interneurons originating from the ganglionic eminence (right, green) migrate tangentially (in the direction of a curved arrow). Thus, ganglionic eminence-targeting in utero electroporation allows for the interneuron-selective expression of foreign genes.

In utero electroporation is an effective method for revealing the function of specific cell populations by selectively inducing the expression of target genes. The opsin-expressing construct is injected into the ventricles of the embryonic brain and electroporated into adjacent cells. Since the cellular lineage of the progenitor cells lining the lateral ventricle varies depending on the embryonic day of the animal, the electroporated cells can be characterized based on the developmental stage [82]. For example, in a developing mouse embryo, electroporation at embryonic day 12.5 (i.e., E12.5), 13.5, or 14.5 results in the expression of target genes in pyramidal neurons at cortical layers V/VI, IV, or II/III, respectively [53,57,60]. Embryonic day-specific in utero electroporation has also been performed in the hippocampus [83]. This targeted cortical layer-specific expression can be explained by the ‘inside-out’ migration pattern of neural precursor cells [84,85]; cells that migrate early in development become neurons in deep layers (closer to cortical layers V/VI), while cells that migrate later in development become neurons in superficial layers (closer to cortical layers II/III) (Figure 4a).

The same technique can be applied to promote the expression of transgenes in interneurons. Interneurons originate in the ganglionic eminence and migrate to the cerebral cortex (Figure 4b). Borrell et al. demonstrated that by targeting the ganglionic eminence, in utero electroporation enabled the selective induction of gene expression in interneurons [86]. In the electroporated adult brain, labeled interneurons can be found in various brain regions, including the neocortex, olfactory bulb, and hippocampus. Borrell et al. also showed that electroporated interneurons were properly integrated into neural networks and exhibited normal electrophysiological properties [86]. Although transgenic animals are normally used to study the role of interneurons, in utero electroporation is an alternative technique for characterizing the developmental and functional properties of interneurons in various regions.

Moreover, changing the time of electroporation enables the transfection of not only neural progenitor cells but also astrocytes. For instance, when rat embryos were electroporated at E18, transfection of astrocytes in the neocortex was observed [87].

## 6. Combining In Utero Electroporation and Optogenetics

In utero electroporation allows target genes to be expressed in the region- and cell type-specific manners. Including opsin-coding genes in the vector plasmids allows the expression of opsins in specific neurons. This technique enables optical control of neuronal activity [88,89] and imaging of neuronal populations [90,91,92]. Here, we describe how in utero electroporation has been used to scrutinize neural circuitry, neural pathways, and animal behavior.

### 6.1. Neural Circuitry

In neural circuits, information is exchanged between different neurons through local and long-distance connections. One way to elucidate the complexity of this neural circuitry is to express opsins in cell- and region-specific manners and manipulate these opsins with optical stimulation. By taking advantage of the cell type- and region-specificity of in utero electroporation, a variety of connections in neural circuits have been scrutinized.

Combining whole-cell recording of synaptic currents with the photostimulation of electroporated channelrhodopsin-2 (ChR2)-positive neurons has allowed mapping of the circuits between presynaptic and postsynaptic neurons. This technique, known as ChR2-assisted circuit mapping (CRACM) [91,93], can be used to identify presynaptic and postsynaptic neurons based on ChR2 expression and whole-cell recordings, respectively. Petreanu et al. used this technique to map long-range callosal projections from layers II/III in the somatosensory cortex and revealed that axons in layers II/III projected to neurons in layers V, II/III, and VI, but not layer IV, in the ipsilateral and contralateral cortices [91]. Furthermore, in both hemispheres, projections from layers II/III to layer V were found to be stronger than those from layers II/III to layers II/III, suggesting that layer specificity may be the same for local and long-range cortical projections. In another study, Petreanu et al. used CRACM to investigate subcellular local connectivity, analyzing projections from thalamic nuclei, the motor cortex, and local excitatory neurons to pyramidal neurons in the mouse somatosensory cortex [93]. They discovered that individual inputs to dendritic arborizations of layer III pyramidal neurons were aligned in a monotonic pattern, while different inputs to layer V pyramidal neurons separately targeted the apical and basal domains of dendrites.

In a similar manner, Adesnik et al. analyzed horizontal projections in cortical domains [94]. In utero electroporation was used to express ChR2 in layers II/III of the somatosensory cortex. The selective activation of horizontally projecting neurons revealed that horizontal projection suppressed activity in the superficial layers and conversely activated deeper output layers.

Bitzenhofer et al. used in utero electroporation to promote ChR2 expression in pyramidal neurons in layers II/III or layers V/VI. They photoactivated ChR2-transfected pyramidal cells with blue light and observed the resulting changes in network oscillations [95]. They showed that the activation of layer II/III pyramidal cells drove frequency-specific spikes and enhanced network oscillations in the β-γ frequency range. On the other hand, the activation of layer V/VI pyramidal cells did not lead to any specificity in activating network oscillations in either frequency range. These results indicate that the entrainment of prefrontal network oscillations to fast rhythms depends on the activation of layer II/III pyramidal cells.

In utero electroporation has also been used to analyze network plasticity in the neocortex. Lourenço et al. found that layer V pyramidal neurons in the mouse barrel cortex modulate information processing in the cortex via perisomatic inhibitory synaptic plasticity [25]. In utero electroporation was used to express ChR2 in layer II/III pyramidal neurons in the mouse barrel cortex. The activation of layer II/III neurons resulted in robust feed-forward inhibition (FFI) of layer V pyramidal neurons via parvalbumin-expressing basket cells. Furthermore, after inducing long-term potentiation of inhibition (LTPi), the facilitation of the excitability of layer V pyramidal neurons triggered by layer II/III activation was greatly reduced. They also found that bursts inducing LTPi-FFI enhanced the temporal relationship between pyramidal neuron spikes and γ-oscillations. These results indicated that the plasticity of parvalbumin-expressing basket cell-dependent periinhibition enabled the strong regulation of single pyramidal neurons at the single-cell and network levels.

Layer specificity in the neocortex has also been used to identify the source of spontaneous low-frequency oscillatory dynamics in the mammalian cortex [96]. Beltramo et al. used viral vectors and in utero electroporation to promote the expression of archaerhodopsin/halorhodopsin and ChR2 in layers V and II/III pyramidal neurons; note that archaerhodopsin-expressing or halorhodopsin-expressing neurons are transiently hyperpolarized by photostimulation. The activation and suppression of specific layers revealed that the activation of layer V pyramidal neurons was sufficient and necessary for generating recurrent low-frequency network oscillations [96].

### 6.2. Developmental and Neonatal Neural Pathways

Wide patterning of brain regions occurs in the early days of embryonic development. Therefore, electroporation into embryos performed as early as at E9.5 allows researchers to study the development of region-wide interactions from early embryonic stages.

Ahlbeck et al. applied in utero electroporation to study the interaction between the hippocampus and neocortex in neonatal mice [97]. They specifically transfected ChR2 into pyramidal neurons in the CA1 area of either the dorsal or intermediate/ventral hippocampus and performed region-specific activation of the hippocampal pyramidal neurons. Photoactivation of CA1 pyramidal neurons, combined with simultaneous recording of local field potentials in the prelimbic subdivision of the prefrontal cortex, revealed that pyramidal neurons in the intermediate/ventral hippocampus, but not the dorsal hippocampus, brought about broad activation of local prefrontal circuits in the neonatal brain [97].

The development of extracellular oscillations in the cerebral cortex has been studied by exploiting the advantages of in utero electroporation, allowing neuronal populations to be manipulated in a layer-specific manner. For example, Bitzenhofer et al. analyzed the origin of gamma oscillations in the developing mouse brain [98]; gamma oscillations are a prominent activity pattern in the cerebral cortex [99]. Using extracellular recordings from early postnatal mice and photostimulation of transfected pyramidal neurons, Bitzenhofer et al. investigated how gamma rhythms were generated during postnatal development [98]. First, fast rhythmic activity in the prefrontal cortex became prominent during the second week after birth. Subsequently, gamma oscillatory activity, approximately 15 Hz at birth, accelerated (in frequency) with age and stabilized in the generally recognized gamma frequency range (i.e., 30–80 Hz) by the postnatal fourth week. This finding was confirmed by photostimulation of pyramidal cells in layers II/III. They also showed similar temporal dynamics in the maturation of fast-spiking interneurons. These findings shed light upon the developmental process of gamma activity in the prefrontal cortex.

In addition, in utero electroporation can be used to study neuropsychiatric pathologies that emerge during development. Bitzenhofer et al. analyzed the effect of disturbed neuronal activity during various developmental stages by transiently increasing the network activity of layer II/III pyramidal neurons in developing mouse brains [100]. In this study, ChR2 was expressed in layer II/III pyramidal neurons in the medial prefrontal cortex using in utero electroporation. Evoking the network activity of postnatal 7- to 11-day-old mice increased the number of prematurely grown dendrites in layer II/III pyramidal neurons. In addition, the gamma power and network synchrony were reduced in the brains of transiently stimulated adult mice. Transient photostimulation also disrupted the excitation-to-inhibition balance, resulting in stronger network inhibition.

### 6.3. Animal Behavior

To reveal a causal relationship between the activation of specific groups of neurons and animal behavior, either excitation or inhibition of the target population of neurons in a specific region can be used. To accomplish this, electrical microstimulation via locally placed electrodes is widely used [101,102,103]. However, this method does not allow the cell types activated by this stimulation to be distinguished. This issue can be overcome by the use of optical stimulation, which acts only on opsin-expressing neurons.

Since in utero electroporation can be used to confine the expression of opsins to specific layers of the cortex, it is possible to control the number and type of neurons excited by photostimulation with optogenetics. Therefore, in utero electroporation is a useful method for analyzing the number of neurons required for perception [89].

## 7. Possible Future Directions

### 7.1. Dual in Utero Electroporation

As described above, in utero electroporation is advantageous in terms of the region-, layer-, and cell type-specific manipulation of neurons. To further dissect the establishment and functions of neuronal networks, it is essential to understand the connectivity and interactions between multiple subsets of neuronal populations. A recent method called dual in utero electroporation serves as an effective tool to target multiple populations of neurons within the same circuit in a spatially and temporally specific manner [104,105,106]. This method has been developed to label or genetically manipulate different subtypes of neurons in different locations. For example, electroporating a plasmid vector containing different fluorescent proteins on different embryonic days will enable researchers to separately label specific subtypes of neurons with distinct spatial and temporal origins [104,105]. Combined with emerging opsins that have a nonoverlapping range of spectral sensitivities [107,108,109], it is possible to separately manipulate multiple subtypes of neurons by expressing opsins with different spectral sensitivities [110,111]. In utero electroporation enables the transfection of DNA into neurons at various developmental stages, in which interventions with viral techniques are difficult. Thus, in utero electroporation may reveal how the activities of different subpopulations of neurons affect the development of cortical networks. Furthermore, region-selective transfection, without the need for genetic markers, can reveal interactions between the same type of neurons with different temporal or spatial origins.

### 7.2. Mosaic and Sparse Labeling of Neurons Using Inducible Gene Targeting

In utero electroporation enables researchers to transfect DNA into a minor subpopulation of neurons [56,112,113]. This approach should be beneficial from electrophysiological and optical standpoints because it is possible to separately stimulate transfected and neighboring nontransfected neurons as a target and control, respectively. In addition, combining an inducible gene targeting system with in utero electroporation enables more precise control of sparse expression of the target transgene. One example is to electroporate Cre-encoding vectors into neurons containing loxP-flanked genes [114]. As another option, the coelectroporation of inducible plasmid-containing genes flanked with loxP sites, and vector-encoding Cre recombinase enables researchers to control the sparseness of expression of transgenes by adjusting the ratio of inducible plasmid and Cre recombinase-encoding vector [114,115,116]. The advantage of this method over simply electroporating low concentrations of plasmid vectors is that electroporated neurons have enough transgene-encoding vectors to induce normal gene expression. This method would enable researchers to sparsely label the target neurons and would further allow the manipulation of sparsely distributed neurons if implemented to express opsins [117]. Furthermore, even single neurons have an impact on sensory perception [118], and single neuronal activities are (partially) influenced by ion channels and ion flow. Sparse in utero electroporation can also be used to label single neurons for live imaging [115]. The expression of opsins or other ion channels using in utero electroporation and spatiotemporally restricted photostimulation can reveal which types of ion channels are required and sufficient for sensory perception even at the single-cell level.

### 7.3. Targeting Astrocytes and Oligodendrocytes

The drawback of standard in utero electroporation is that electroporated transgenes remain episomal and thus are lost during cell division, suggesting that transgenes are not expressed in proliferating cells, such as cells in a neural lineage. However, combining the binary piggyBac transposon system with in utero electroporation enables the stable expression of transgenes in cells in the neural lineage, such as radial glia [119,120,121,122]. Using this technique, researchers can induce the expression of transgenes even in proliferating cells such as astrocytes and oligodendrocytes [120,121]. Researchers in these previous studies using the transposon system adopted this method to knockdown or overexpress target genes. In recent years, optogenetics has been applied to elucidate physiological and pathological functions of astrocytes [123,124,125,126]. We believe that the combination of the piggyBac transposon system with in utero electroporation can also be used to promote the expression of opsins to photostimulate astrocytes and oligodendrocytes.

In this light, the combination of a variety of in utero electroporation-based techniques (i.e., 1. the multiexpression of transgenes in different regions, 2. the sparse expression of transgenes with inducible methods, and 3. techniques to target the neural lineage by the transposon system) with optogenetics will open new avenues for the theoretical and experimental investigations to answer fundamental questions in neuroscience.

## References

[1] Deisseroth K., Feng G., Majewska A.K., Miesenbock G., Ting A., Schnitzer M.J. (2006). Next-generation optical technologies for illuminating genetically targeted brain circuits. J. Neurosci..

[2] Beck S., Yu-Strzelczyk J., Pauls D., Constantin O.M., Gee C.E., Ehmann N., Kittel R.J., Nagel G., Gao S. (2018). Synthetic light-activated ion channels for optogenetic activation and inhibition. Front. Neurosci..

[3] Boyden E.S., Zhang F., Bamberg E., Nagel G., Deisseroth K. (2005). Millisecond-timescale, genetically targeted optical control of neural activity. Nat. Neurosci..

[4] Packer A.M., Peterka D.S., Hirtz J.J., Prakash R., Deisseroth K., Yuste R. (2012). Two-photon optogenetics of dendritic spines and neural circuits. Nat. Methods.

[5] Yang W., Carrillo-Reid L., Bando Y., Peterka D.S., Yuste R. (2018). Simultaneous two-photon imaging and two-photon optogenetics of cortical circuits in three dimensions. Elife.

[6] Engelhard C., Chizhov I., Siebert F., Engelhard M. (2018). Microbial halorhodopsins: Light-driven chloride pumps. Chem. Rev..

[7] Chernov M.M., Friedman R.M., Chen G., Stoner G.R., Roe A.W. (2018). Functionally specific optogenetic modulation in primate visual cortex. Proc. Natl. Acad. Sci. USA.

[8] Toso A., Fassihi A., Paz L., Pulecchi F., Diamond M.E. (2021). A sensory integration account for time perception. PLoS Comput. Biol..

[9] Rikhye R.V., Yildirim M., Hu M., Breton-Provencher V., Sur M. (2021). Reliable sensory processing in mouse visual cortex through cooperative interactions between somatostatin and parvalbumin interneurons. J. Neurosci..

[10] Saumweber T., Rohwedder A., Schleyer M., Eichler K., Chen Y., Aso Y., Cardona A., Eschbach C., Kobler O., Voigt A. (2018). Functional architecture of reward learning in mushroom body extrinsic neurons of larval Drosophila. Nat. Commun..

[11] Lyutova R., Selcho M., Pfeuffer M., Segebarth D., Habenstein J., Rohwedder A., Frantzmann F., Wegener C., Thum A.S., Pauls D. (2019). Reward signaling in a recurrent circuit of dopaminergic neurons and peptidergic Kenyon cells. Nat. Commun..

[12] Fan Z., Wu B., Wu G., Yao J., Li X., Hu K., Zhou Z., Sui J. (2019). Optogenetic inhibition of ventral hippocampal neurons alleviates associative motor learning dysfunction in a rodent model of schizophrenia. PLoS ONE.

[13] Jarrin S., Pandit A., Roche M., Finn D.P. (2020). Differential role of anterior cingulate cortical glutamatergic neurons in pain-related aversion learning and nociceptive behaviors in male and female rats. Front. Behav. Neurosci..

[14] Zemelman B.V., Lee G.A., Ng M., Miesenböck G. (2002). Selective photostimulation of genetically ChARGed neurons. Neuron.

[15] Nagel G., Szellas T., Huhn W., Kateriya S., Adeishvili N., Berthold P., Ollig D., Hegemann P., Bamberg E. (2003). Channelrhodopsin-2, a directly light-gated cation-selective membrane channel. Proc. Natl. Acad. Sci. USA.

[16] Zhang F., Prigge M., Beyrière F., Tsunoda S.P., Mattis J., Yizhar O., Hegemann P., Deisseroth K. (2008). Red-shifted optogenetic excitation: A tool for fast neural control derived from Volvox carteri. Nat. Neurosci..

[17] Mattis J., Tye K.M., Ferenczi E.A., Ramakrishnan C., O’Shea D.J., Prakash R., Gunaydin L.A., Hyun M., Fenno L.E., Gradinaru V. (2012). Principles for applying optogenetic tools derived from direct comparative analysis of microbial opsins. Nat. Methods.

[18] Broyles C., Robinson P., Daniels M. (2018). Fluorescent, bioluminescent, and optogenetic approaches to study excitable physiology in the single cardiomyocyte. Cells.

[19] Govorunova E.G., Sineshchekov O.A., Janz R., Liu X., Spudich J.L. (2015). Natural light-gated anion channels: A family of microbial rhodopsins for advanced optogenetics. Science.

[20] Kim Y.S., Kato H.E., Yamashita K., Ito S., Inoue K., Ramakrishnan C., Fenno L.E., Evans K.E., Paggi J.M., Dror R.O. (2018). Crystal structure of the natural anion-conducting channelrhodopsin GtACR1. Nature.

[21] Alberio L., Locarno A., Saponaro A., Romano E., Bercier V., Albadri S., Simeoni F., Moleri S., Pelucchi S., Porro A. (2018). A light-gated potassium channel for sustained neuronal inhibition. Nat. Methods.

[22] Arrenberg A.B., Stainier D.Y.R., Baier H., Huisken J. (2010). Optogenetic control of cardiac function. Science.

[23] Bernal Sierra Y.A., Rost B.R., Pofahl M., Fernandes A.M., Kopton R.A., Moser S., Holtkamp D., Masala N., Beed P., Tukker J.J. (2018). Potassium channel-based optogenetic silencing. Nat. Commun..

[24] Adesnik H. (2018). Layer-specific excitation/inhibition balances during neuronal synchronization in the visual cortex. J. Physiol..

[25] Lourenço J., De Stasi A.M., Deleuze C., Bigot M., Pazienti A., Aguirre A., Giugliano M., Ostojic S., Bacci A. (2020). Modulation of coordinated activity across cortical layers by plasticity of inhibitory synapses. Cell Rep..

[26] Otsuka T., Kawaguchi Y. (2021). Pyramidal cell subtype-dependent cortical oscillatory activity regulates motor learning. Commun. Biol..

[27] Fenno L.E., Deisseroth K., Weber B., Helmchen F. (2014). Neocortical Circuit Interrogation with Optogenetics. Optical Imaging of Neocortical Dynamics.

[28] Ainsworth C. (2015). Agriculture: A new breed of edits. Nature.

[29] Urnov F.D., Miller J.C., Lee Y.-L., Beausejour C.M., Rock J.M., Augustus S., Jamieson A.C., Porteus M.H., Gregory P.D., Holmes M.C. (2005). Highly efficient endogenous human gene correction using designed zinc-finger nucleases. Nature.

[30] Blattner G., Cavazza A., Thrasher A.J., Turchiano G. (2020). Gene editing and genotoxicity: Targeting the off-targets. Front. Genome Ed..

[31] Liu Q., Segal D.J., Ghiara J.B., Barbas C.F. (1997). Design of polydactyl zinc-finger proteins for unique addressing within complex genomes. Proc. Natl. Acad. Sci. USA.

[32] Beerli R.R., Barbas C.F. (2002). Engineering polydactyl zinc-finger transcription factors. Nat. Biotechnol..

[33] Beerli R.R., Dreier B., Barbas C.F. (2000). Positive and negative regulation of endogenous genes by designed transcription factors. Proc. Natl. Acad. Sci. USA.

[34] Bhakta M.S., Henry I.M., Ousterout D.G., Das K.T., Lockwood S.H., Meckler J.F., Wallen M.C., Zykovich A., Yu Y., Leo H. (2013). Highly active zinc-finger nucleases by extended modular assembly. Genome Res..

[35] Watanabe Y., Okuya K., Takada Y., Kinoshita M., Yokoi S., Chisada S., Kamei Y., Tatsukawa H., Yamamoto N., Abe H. (2020). Gene disruption of medaka (*Oryzias latipes*) orthologue for mammalian tissue-type transglutaminase (TG2) causes movement retardation. J. Biochem..

[36] Bogdanove A.J., Voytas D.F. (2011). TAL effectors: Customizable proteins for DNA targeting. Science.

[37] Yokoi S., Okuyama T., Kamei Y., Naruse K., Taniguchi Y., Ansai S., Kinoshita M., Young L.J., Takemori N., Kubo T. (2015). An essential role of the arginine vasotocin system in mate-guarding behaviors in triadic relationships of medaka fish (*Oryzias latipes*). PLoS Genet..

[38] Christian M., Cermak T., Doyle E.L., Schmidt C., Zhang F., Hummel A., Bogdanove A.J., Voytas D.F. (2010). Targeting DNA double-strand breaks with TAL effector nucleases. Genetics.

[39] Mussolino C., Morbitzer R., Lütge F., Dannemann N., Lahaye T., Cathomen T. (2011). A novel TALE nuclease scaffold enables high genome editing activity in combination with low toxicity. Nucleic Acids Res..

[40] Miller J.C., Tan S., Qiao G., Barlow K.A., Wang J., Xia D.F., Meng X., Paschon D.E., Leung E., Hinkley S.J. (2011). A TALE nuclease architecture for efficient genome editing. Nat. Biotechnol..

[41] Reyon D., Tsai S.Q., Khayter C., Foden J.A., Sander J.D., Joung J.K. (2012). FLASH assembly of TALENs for high-throughput genome editing. Nat. Biotechnol..

[42] Jinek M., Chylinski K., Fonfara I., Hauer M., Doudna J.A., Charpentier E. (2012). A programmable dual-RNA–guided DNA endonuclease in adaptive bacterial immunity. Science.

[43] Hsu P.D., Lander E.S., Zhang F. (2014). Development and applications of CRISPR-Cas9 for genome engineering. Cell.

[44] Cong L., Ran F.A., Cox D., Lin S., Barretto R., Habib N., Hsu P.D., Wu X., Jiang W., Marraffini L.A. (2013). Multiplex genome engineering using CRISPR/Cas systems. Science.

[45] Mali P., Yang L., Esvelt K.M., Aach J., Guell M., DiCarlo J.E., Norville J.E., Church G.M. (2013). RNA-guided human genome engineering via Cas9. Science.

[46] Davidson B.L., Breakefield X.O. (2003). Viral vectors for gene delivery to the nervous system. Nat. Rev. Neurosci..

[47] Tervo D.G.R., Hwang B.-Y., Viswanathan S., Gaj T., Lavzin M., Ritola K.D., Lindo S., Michael S., Kuleshova E., Ojala D. (2016). A designer AAV variant permits efficient retrograde access to projection neurons. Neuron.

[48] Mebatsion T., Schnell M.J., Cox J.H., Finke S., Conzelmann K.K. (1996). Highly stable expression of a foreign gene from rabies virus vectors. Proc. Natl. Acad. Sci. USA.

[49] Mody P.H., Pathak S., Hanson L.K., Spencer J.V. (2020). Herpes simplex virus: A versatile tool for insights into evolution, gene delivery, and tumor immunotherapy. Virol. Res. Treat..

[50] Mohammadzadeh Y., Gholami S., Rasouli N., Sarrafzadeh S., Seyed Tabib N.S., Samiee Aref M.H., Abdoli A., Biglari P., Fotouhi F., Farahmand B. (2017). Introduction of cationic virosome derived from vesicular stomatitis virus as a novel gene delivery system for sf9 cells. J. Liposome Res..

[51] Fukuchi-Shimogori T., Grove E.A. (2001). Neocortex patterning by the secreted signaling molecule FGF8. Science.

[52] Mizuno H., Hirano T., Tagawa Y. (2007). Evidence for activity-dependent cortical wiring: Formation of interhemispheric connections in neonatal mouse visual cortex requires projection neuron activity. J. Neurosci..

[53] Meyer-Dilhet G., Courchet J. (2020). In utero cortical electroporation of plasmids in the mouse embryo. STAR Protoc..

[54] Comer A.L., Sriram B., Yen W.W., Cruz-Martín A. (2020). A pipeline using bilateral in utero electroporation to interrogate genetic influences on rodent behavior. J. Vis. Exp..

[55] Tabata H., Nakajima K. (2001). Efficient in utero gene transfer system to the developing mouse brain using electroporation: Visualization of neuronal migration in the developing cortex. Neuroscience.

[56] Shimogori T., Ogawa M. (2008). Gene application with in utero electroporation in mouse embryonic brain. Dev. Growth Differ..

[57] Matsui A., Yoshida A.C., Kubota M., Ogawa M., Shimogori T. (2011). Mouse in utero electroporation: Controlled spatiotemporal gene transfection. J. Vis. Exp..

[58] Dal Maschio M., Ghezzi D., Bony G., Alabastri A., Deidda G., Brondi M., Sato S.S., Zaccaria R.P., Di Fabrizio E., Ratto G.M. (2012). High-performance and site-directed in utero electroporation by a triple-electrode probe. Nat. Commun..

[59] Bullmann T., Arendt T., Frey U., Hanashima C. (2015). A transportable, inexpensive electroporator for in utero electroporation. Dev. Growth Differ..

[60] Kamiya A. (2009). Animal models for schizophrenia via in utero gene transfer: Understanding roles for genetic susceptibility factors in brain development. Prog. Brain Res..

[61] Kalebic N., Langen B., Helppi J., Kawasaki H., Huttner W.B. (2020). In vivo targeting of neural progenitor cells in ferret neocortex by in utero electroporation. J. Vis. Exp..

[62] Ohmura N., Kawasaki K., Satoh T., Hata Y. (2015). In vivo electroporation to physiologically identified deep brain regions in postnatal mammals. Brain Struct. Funct..

[63] Taniguchi Y., Young-Pearse T., Sawa A., Kamiya A. (2012). In utero electroporation as a tool for genetic manipulation in vivo to study psychiatric disorders: From genes to circuits and behaviors. Neurosci..

[64] Fietz S.A., Huttner W.B. (2011). Cortical progenitor expansion, self-renewal and neurogenesis—a polarized perspective. Curr. Opin. Neurobiol..

[65] Kawaguchi A., Ikawa T., Kasukawa T., Ueda H.R., Kurimoto K., Saitou M., Matsuzaki F. (2008). Single-cell gene profiling defines differential progenitor subclasses in mammalian neurogenesis. Development.

[66] Hansen D.V., Lui J.H., Parker P.R.L., Kriegstein A.R. (2010). Neurogenic radial glia in the outer subventricular zone of human neocortex. Nature.

[67] Nakanishi H., Higuchi Y., Kawakami S., Yamashita F., Hashida M. (2010). piggyBac transposon-mediated long-term gene expression in mice. Mol. Ther..

[68] Ding S., Wu X., Li G., Han M., Zhuang Y., Xu T. (2005). Efficient transposition of the piggyBac (PB) transposon in mammalian cells and mice. Cell.

[69] Neumann E., Schaefer-Ridder M., Wang Y., Hofschneider P.H. (1982). Gene transfer into mouse lyoma cells by electroporation in high electric fields. EMBO J..

[70] Weaver J.C. (2003). Electroporation of biological membranes from multicellular to nano scales. IEEE Trans. Dielectr. Electr. Insul..

[71] Mir L.M., Moller P.H., André F., Gehl J. (2005). Electric pulse-mediated gene delivery to various animal tissues. Adv. Genet..

[72] Szczurkowska J., Cwetsch A.W., dal Maschio M., Ghezzi D., Ratto G.M., Cancedda L. (2016). Targeted in vivo genetic manipulation of the mouse or rat brain by in utero electroporation with a triple-electrode probe. Nat. Protoc..

[73] Šatkauskas S., André F., Bureau M.F., Scherman D., Miklavčič D., Mir L.M. (2005). Electrophoretic component of electric pulses determines the efficacy of in vivo DNA electrotransfer. Hum. Gene Ther..

[74] Rols M.-P., Li S. (2008). Mechanism by which electroporation mediates DNA migration and entry into cells and targeted tissues. Methods in Molecular Biology.

[75] Klenchin V.A., Sukharev S.I., Serov S.M., Chernomordik L.V., Chizmadzhev Y. (1991). Electrically induced DNA uptake by cells is a fast process involving DNA electrophoresis. Biophys. J..

[76] Sukharev S.I., Klenchin V.A., Serov S.M., Chernomordik L.V., Chizmadzhev Y. (1992). Electroporation and electrophoretic DNA transfer into cells. The effect of DNA interaction with electropores. Biophys. J..

[77] Ito H., Morishita R., Iwamoto I., Nagata K. (2014). Establishment of an in vivo electroporation method into postnatal newborn neurons in the dentate gyrus. Hippocampus.

[78] Baumgart J., Baumgart N. (2016). Cortex-, hippocampus-, thalamus-, hypothalamus-, lateral septal nucleus- and striatum-specific in utero electroporation in the C57BL/6 mouse. J. Vis. Exp..

[79] Pacary E., Haas M.A., Wildner H., Azzarelli R., Bell D.M., Abrous D.N., Guillemot F. (2012). Visualization and genetic manipulation of dendrites and spines in the mouse cerebral cortex and hippocampus using in utero electroporation. J. Vis. Exp..

[80] Bai J., Ramos R.L., Paramasivam M., Siddiqi F., Ackman J.B., LoTurco J.J. (2008). The role of DCX and LIS1 in migration through the lateral cortical stream of developing forebrain. Dev. Neurosci..

[81] Saito T., Nakatsuji N. (2001). Efficient gene transfer into the embryonic mouse brain using in vivo electroporation. Dev. Biol..

[82] Hand R., Polleux F. (2011). Neurogenin2 regulates the initial axon guidance of cortical pyramidal neurons projecting medially to the corpus callosum. Neural Dev..

[83] Navarro-Quiroga I., Chittajallu R., Gallo V., Haydar T.F. (2007). Long-term, selective gene expression in developing and adult hippocampal pyramidal neurons using focal in utero electroporation. J. Neurosci..

[84] Rakic P. (1971). Guidance of neurons migrating to the fetal monkey neocortex. Brain Res..

[85] Greig L.C., Woodworth M.B., Galazo M.J., Padmanabhan H., Macklis J.D. (2013). Molecular logic of neocortical projection neuron specification, development and diversity. Nat. Rev. Neurosci..

[86] Borrell V., Yoshimura Y., Callaway E.M. (2005). Targeted gene delivery to telencephalic inhibitory neurons by directional in utero electroporation. J. Neurosci. Methods.

[87] LoTurco J., Manent J.-B., Sidiqi F. (2009). New and improved tools for in utero electroporation studies of developing cerebral cortex. Cereb. Cortex.

[88] Bitzenhofer S.H., Ahlbeck J., Hanganu-Opatz I.L. (2017). Methodological approach for optogenetic manipulation of neonatal neuronal networks. Front. Cell. Neurosci..

[89] Huber D., Petreanu L., Ghitani N., Ranade S., Hromádka T., Mainen Z., Svoboda K. (2008). Sparse optical microstimulation in barrel cortex drives learned behaviour in freely moving mice. Nature.

[90] Gee J.M., Gibbons M.B., Taheri M., Palumbos S., Morris S.C., Smeal R.M., Flynn K.F., Economo M.N., Cizek C.G., Capecchi M.R. (2015). Imaging activity in astrocytes and neurons with genetically encoded calcium indicators following in utero electroporation. Front. Mol. Neurosci..

[91] Petreanu L., Huber D., Sobczyk A., Svoboda K. (2007). Channelrhodopsin-2–assisted circuit mapping of long-range callosal projections. Nat. Neurosci..

[92] Rolotti S.V., Blockus H., Sparks F.T., Priestley J.B., Losonczy A. (2022). Reorganization of CA1 dendritic dynamics by hippocampal sharp-wave ripples during learning. Neuron.

[93] Petreanu L., Mao T., Sternson S.M., Svoboda K. (2009). The subcellular organization of neocortical excitatory connections. Nature.

[94] Adesnik H., Scanziani M. (2010). Lateral competition for cortical space by layer-specific horizontal circuits. Nature.

[95] Bitzenhofer S.H., Ahlbeck J., Wolff A., Wiegert J.S., Gee C.E., Oertner T.G., Hanganu-Opatz I.L. (2017). Layer-specific optogenetic activation of pyramidal neurons causes beta–gamma entrainment of neonatal networks. Nat. Commun..

[96] Beltramo R., D’Urso G., Dal Maschio M., Farisello P., Bovetti S., Clovis Y., Lassi G., Tucci V., De Pietri Tonelli D., Fellin T. (2013). Layer-specific excitatory circuits differentially control recurrent network dynamics in the neocortex. Nat. Neurosci..

[97] Ahlbeck J., Song L., Chini M., Bitzenhofer S.H., Hanganu-Opatz I.L. (2018). Glutamatergic drive along the septo-temporal axis of hippocampus boosts prelimbic oscillations in the neonatal mouse. eLife.

[98] Bitzenhofer S.H., Pöpplau J.A., Hanganu-Opatz I. (2020). Gamma activity accelerates during prefrontal development. eLife.

[99] Buzsáki G., Wang X.-J. (2012). Mechanisms of gamma oscillations. Annu. Rev. Neurosci..

[100] Bitzenhofer S.H., Pöpplau J.A., Chini M., Marquardt A., Hanganu-Opatz I.L. (2021). A transient developmental increase in prefrontal activity alters network maturation and causes cognitive dysfunction in adult mice. Neuron.

[101] Callier T., Brantly N.W., Caravelli A., Bensmaia S.J. (2020). The frequency of cortical microstimulation shapes artificial touch. Proc. Natl. Acad. Sci. USA.

[102] Salzman C.D., Britten K.H., Newsome W.T. (1990). Cortical microstimulation influences perceptual judgements of motion direction. Nature.

[103] Voigt M.B., Yusuf P.A., Kral A. (2018). Intracortical microstimulation modulates cortical induced responses. J. Neurosci..

[104] Zhang L., Getz S.A., Bordey A. (2022). Dual in utero electroporation in mice to manipulate two specific neuronal populations in the developing cortex. Front. Bioeng. Biotechnol..

[105] Mateos-White I., Fabra-Beser J., de Agustín-Durán D., Gil-Sanz C. (2020). Double in utero electroporation to target temporally and spatially separated cell populations. J. Vis. Exp..

[106] Taylor R.J., Carrington J., Gerlach L.R., Taylor K.L., Richters K.E., Dent E.W. (2020). Double UP: A dual color, internally controlled platform for in utero knockdown or overexpression. Front. Mol. Neurosci..

[107] Duebel J., Marazova K., Sahel J.-A. (2015). Optogenetics. Curr. Opin. Ophthalmol..

[108] Han X., Boyden E.S. (2007). Multiple-color optical activation, silencing, and desynchronization of neural activity, with single-spike temporal resolution. PLoS ONE.

[109] Wietek J., Beltramo R., Scanziani M., Hegemann P., Oertner T.G., Wiegert J.S. (2015). An improved chloride-conducting channelrhodopsin for light-induced inhibition of neuronal activity in vivo. Sci. Rep..

[110] Kampasi K., English D.F., Seymour J., Stark E., McKenzie S., Vöröslakos M., Buzsáki G., Wise K.D., Yoon E. (2018). Dual color optogenetic control of neural populations using low-noise, multishank optoelectrodes. Microsyst. Nanoeng..

[111] Marshel J.H., Kim Y.S., Machado T.A., Quirin S., Benson B., Kadmon J., Raja C., Chibukhchyan A., Ramakrishnan C., Inoue M. (2019). Cortical layer–specific critical dynamics triggering perception. Science.

[112] Inoue T., Krumlauf R. (2001). An impulse to the brain—using in vivo electroporation. Nat. Neurosci..

[113] Rahim A.A., Wong A.M.S., Buckley S.M.K., Chan J.K.Y., David A.L., Cooper J.D., Coutelle C., Peebles D.M., Waddington S.N. (2010). In utero gene transfer to the mouse nervous system. Biochem. Soc. Trans..

[114] Bland K.M., Casey Z.O., Handwerk C.J., Holley Z.L., Vidal G.S. (2017). Inducing Cre-lox recombination in mouse cerebral cortex through in utero electroporation. J. Vis. Exp..

[115] Schohl A., Chorghay Z., Ruthazer E.S. (2020). A simple and efficient method for visualizing individual cells in vivo by Cre-mediated single-cell labeling by electroporation (CREMSCLE). Front. Neural Circuits.

[116] Khoo A.T.T., Kim P.J., Kim H.M., Je H.S. (2020). Neural circuit analysis using a novel intersectional split intein-mediated split-Cre recombinase system. Mol. Brain.

[117] Sun Y.J., Espinosa J.S., Hoseini M.S., Stryker M.P. (2019). Experience-dependent structural plasticity at pre- and postsynaptic sites of layer 2/3 cells in developing visual cortex. Proc. Natl. Acad. Sci. USA.

[118] Houweling A.R., Brecht M. (2008). Behavioural report of single neuron stimulation in somatosensory cortex. Nature.

[119] Chen F., LoTurco J. (2012). A method for stable transgenesis of radial glia lineage in rat neocortex by piggyBac mediated transposition. J. Neurosci. Methods.

[120] Siddiqi F., Chen F., Aron A.W., Fiondella C.G., Patel K., LoTurco J.J. (2014). Fate mapping by piggyBac transposase reveals that neocortical GLAST+ progenitors generate more astrocytes than Nestin+ progenitors in rat neocortex. Cereb. Cortex.

[121] Dinh Duong T.A., Hoshiba Y., Saito K., Kawasaki K., Ichikawa Y., Matsumoto N., Shinmyo Y., Kawasaki H. (2019). FGF signaling directs the cell fate switch from neurons to astrocytes in the developing mouse cerebral cortex. J. Neurosci..

[122] Hamabe-Horiike T., Kawasaki K., Sakashita M., Ishizu C., Yoshizaki T., Harada S.-I., Ogawa-Ochiai K., Shinmyo Y., Kawasaki H. (2021). Glial cell type-specific gene expression in the mouse cerebrum using the piggyBac system and in utero electroporation. Sci. Rep..

[123] Perea G., Yang A., Boyden E.S., Sur M. (2014). Optogenetic astrocyte activation modulates response selectivity of visual cortex neurons in vivo. Nat. Commun..

[124] Yang F., Liu Y., Tu J., Wan J., Zhang J., Wu B., Chen S., Zhou J., Mu Y., Wang L. (2014). Activated astrocytes enhance the dopaminergic differentiation of stem cells and promote brain repair through bFGF. Nat. Commun..

[125] Mederos S., Hernández-Vivanco A., Ramírez-Franco J., Martín-Fernández M., Navarrete M., Yang A., Boyden E.S., Perea G. (2019). Melanopsin for precise optogenetic activation of astrocyte-neuron networks. Glia.

[126] Gomez J.A., Perkins J.M., Beaudoin G.M., Cook N.B., Quraishi S.A., Szoeke E.A., Thangamani K., Tschumi C.W., Wanat M.J., Maroof A.M. (2019). Ventral tegmental area astrocytes orchestrate avoidance and approach behavior. Nat. Commun..

